# Health influenced by genetics: A first comprehensive analysis of breast cancer high and moderate penetrance susceptibility genes in the Tunisian population

**DOI:** 10.1371/journal.pone.0265638

**Published:** 2022-03-25

**Authors:** Maroua Boujemaa, Najah Mighri, Lotfi Chouchane, Mohamed Samir Boubaker, Sonia Abdelhak, Hamouda Boussen, Yosr Hamdi

**Affiliations:** 1 Laboratory of Biomedical Genomics and Oncogenetics, Institut Pasteur de Tunis, University of Tunis El Manar, Tunis, Tunisia; 2 Department of Genetic Medicine, Weill Cornell Medicine, New York, New York, United States of America; 3 Department of Microbiology and Immunology, Weill Cornell Medicine, New York, New York, United States of America; 4 Laboratory of Genetic Medicine and Immunology, Weill Cornell Medicine-Qatar, Doha, Qatar; 5 Laboratory of Human and Experimental Pathology, Institut Pasteur de Tunis, Tunis, Tunisia; 6 Medical Oncology Department, Abderrahman Mami Hospital, Faculty of Medicine Tunis, University Tunis El Manar, Tunis, Tunisia; University of Chicago, UNITED STATES

## Abstract

Significant advances have been made to understand the genetic basis of breast cancer. High, moderate and low penetrance variants have been identified with inter-ethnic variability in mutation frequency and spectrum. Genome wide association studies (GWAS) are widely used to identify disease-associated SNPs. Understanding the functional impact of these risk-SNPs will help the translation of GWAS findings into clinical interventions. Here we aim to characterize the genetic patterns of high and moderate penetrance breast cancer susceptibility genes and to assess the functional impact of non-coding SNPs. We analyzed *BRCA1/2*, *PTEN*, *STK11*, *TP53*, *ATM*, *BRIP1*, *CHEK2* and *PALB2* genotype data obtained from 135 healthy participants genotyped using Affymetrix Genome-Wide Human SNP-Array 6.0. Haplotype analysis was performed using Haploview.V4.2 and PHASE.V2.1. Population structure and genetic differentiation were assessed using principal component analysis (PCA) and fixation index (FST). Functional annotation was performed using *In Silico* web-based tools including RegulomeDB and VARAdb. Haplotype analysis showed distinct LD patterns with high levels of recombination and haplotype blocks of moderate to small size. Our findings revealed also that the Tunisian population tends to have a mixed origin with European, South Asian and Mexican footprints. Functional annotation allowed the selection of 28 putative regulatory variants. Of special interest were *BRCA1_* rs8176318 predicted to alter the binding sites of a tumor suppressor miRNA hsa-miR-149 and *PALB2_* rs120963 located in tumorigenesis-associated enhancer and predicted to strongly affect the binding of P53. Significant differences in allele frequencies were observed with populations of African and European ancestries for rs8176318 and rs120963 respectively. Our findings will help to better understand the genetic basis of breast cancer by guiding upcoming genome wide studies in the Tunisian population. Putative functional SNPs may be used to develop an efficient polygenic risk score to predict breast cancer risk leading to better disease prevention and management.

## Introduction

Breast cancer is the most common malignancy among women and the first leading cause of cancer death in females worldwide [1]. In Tunisia, it represents 30% of all female malignancies, with an incidence of 30/100000 making it a major public health concern [2, 3]. It is also characterized by an early age at diagnosis, ten years younger than that observed in western countries [4, 5]. About 5–10% of all breast cancer cases can be attributed to hereditary factors and up to 30% of which are due to germline mutations in *BRCA1*/2 genes [6]. Other less frequently altered genes, namely *CDH1*, *PTEN*, *STK11* and *TP53*, have been also found to increase breast cancer risk. Mutations in these genes account for approximately 5% of the familial breast cancer risk [7, 8]. In addition, more frequent, but less penetrant germline mutations have been identified in families with breast cancer in genes such as *CHEK2*, *ATM*, *PALB2*, and *BRIP1* [9]. Breast cancer has a heterogeneous genetic component. The frequency and spectrum of mutations in predisposing genes vary widely among populations. Some mutations are observed in distinct populations and others are ethnic specific due to potential founder effect [10, 11]. This interethnic variability is highly influenced by the genetic background and the genetic structure of a given population that play an important role in disease susceptibility. Indeed, genetic patterns, including linkage disequilibrium (LD) profiles, haplotype structure and allele frequencies, differ from one ethnic group to another. Characterizing these genetic patterns may help to decipher the biological mechanisms behind breast cancer development [12].

Furthermore, Genome-wide association studies (GWAS) have successfully mapped thousands of loci associated with complex human diseases and phenotypes including breast cancer [13, 14]. So far, more than 170 common genetic variants associated with breast cancer risk have been identified. These common variants account for about 18% of the familial relative risk [14].

To translate GWAS findings into biological insights towards clinical applications, it is crucial to understand the functional impact of disease risk SNPs. These latter, often lie in non-coding regions, presumably in regulatory regions, such as distal enhancers, transcription factor (TF) binding sites, and accessible chromatin regions [15].

Various studies have shown that disease-associated variants were considerably enriched in regulatory elements that are widely spread across the human genome. Indeed, several variants for instance rs1198588 (located upstream MIR137HG), rs4442975 *(LOC101928278)* and rs117480515*(TNFAIP3)* have been found to affect the TF binding sites and to regulate gene expression [15]. It was demonstrated also that rs4442975, which is associated with breast cancer, disrupts the recruitment of FOXA1 and interacts with the *IGFBP5* promoter [16]. An increasing number of studies have also shown that genetic variations located within the 3’UTR region may disrupt existing or create new miRNA binding sites which promote disease development and cancer pathogenesis [17].

To this end, and to improve our knowledge on the genetic background and predisposition to breast cancer in the Tunisian population we have used genotype data of 135 healthy Tunisian individuals to characterize the genetic patterns of high and moderate penetrance breast cancer susceptibility genes and to assess the functional impact of non-coding SNPs identified within these genes.

## Materials and methods

### Studied population

The study cohort comprised a total of 135 healthy individuals (103 men and 32 women) originating from distinct regions of Tunisia. No evidence of personal or family history of breast or other malignancies have been detected in these families. Indeed, a detailed family history questionnaire was conducted in order to collect medical information (cancer and genetic diseases) of all family members including first, second- and third-degree relatives or any other family member with personal history of cancer. Accordingly, only subjects with no family history of cancer were selected.

Written informed consent was obtained from all subjects prior to specimen collection. This study was conducted in accordance with the ethical standards of Helsinki declaration and approved by the biomedical ethics committee of Institut Pasteur de Tunis (Tunis, Tunisia-Registration numbers, PV09/06 IRB# 0,000,000,044).

### Genotyping data analysis and SNP selection

Genotyping data of high and moderate penetrance breast cancer susceptibility genes relative to 135 healthy participants previously investigated using Affymetrix Genome-Wide Human SNP Array 6.0 [18]. were analyzed. SNPs detected in each gene of interest namely *BRCA1*, *BRCA2*, *PTEN*, *STK11*, *TP53*, *BRIP1*, *CHEK2* and *PALB2* as well as those flanking 50kb on either side, covering the surrounding regulatory regions, were extracted using PLINK version 1.9 [19]. Variants deviating from the Hardy-Weinberg equilibrium (HW p-value cutoff <10−4) and with less than 75% of genotype data were excluded. A total of 387 variants were considered for further analysis. Genotype data, covering the selected SNPs, of individuals from 8 distinct populations of the 1000 genomes project Phase III were downloaded [20]. using the DataSlicer ensembl’s tool (https://www.ensembl.org/Homo_sapiens/Tools/DataSlicer). The 8 investigated populations are the followings: Utah Residents (CEPH) with Northern and Western European Ancestry (CEU), Han Chinese in Beijing, China (CHB), Gujarati Indian from Houston, Texas (GIH), Japanese in Tokyo, Japan (JPT), Luhya in Webuye, Kenya (LWK), Mexican Ancestry from Los Angeles USA (MXL), Toscani in Italia (TSI), Yoruba in Ibadan, Nigeria (YRI).

### Haplotype analysis

Haploview version 4.2 was used for the analysis of LD patterns and haplotype blocks as well as for the detection of tagging SNPs based on the genotype data of the 135 healthy Tunisian subjects. LD blocks were defined according to the haplotype block definition of Gabriel which defines a pair of SNPs to be in “strong LD” if the one-sided upper 95% confidence bound on D’ is >0.98 and the lower bound is above 0.7 [21].

In each sample, the genotype frequencies for each SNP were checked for consistency between the observed values and those expected from Hardy-Weinberg equilibrium using the same software. We used the program PHASE version 2.1 in order to estimate haplotypes distribution of *BRCA1* and *BRCA2* genes using genotype data of tagging SNPs. PHASE implements a Bayesian statistical method for reconstructing haplotypes from population genotype data [22, 23]. The resulting haplotypes in addition to those of 8 populations (CEU, CHB, GIH, JPT, LWK, MXL, TSI and YRI) of the 1000 genomes project (constructed using PHASE2.1) were aligned with MEGA version 7 [24]. These haplotypes were then used to construct a haplotype network using Network software 5.0.0.1 (http://www.fluxus-engineering.com/netwinfo.htm) in order to assess intrapopulation variability in haplotype distribution. Only haplotypes having frequency >1% were considered for the Haplotype Network analysis.

### Population structure and genetic diversity analysis

Population structure and genetic differentiation were evaluated using principal component analysis (PCA) and fixation index (FST).

PCA was performed using the toolset Plink1.9. The two first PCAs were then plotted using the ggplot2 R package [25]. When performing PCA analysis only SNPs with the following criteria were considered: HWE p-value > 0.0001, have a genotyping call rate of greater than 95%, MAF ≥0.05, and r2 < 0.6. FST-values were calculated using Plink1.9 based on the method of Weir and Cockerham [19]. FST-values range from 0 to 1. High Fst implies a considerable degree of differentiation among populations while values near 0 indicate little genetic subdivision.

### Functional effect prediction

To predict the functional impact of the studied SNPs and to identify functional variants that could contribute to disease risk, several *In Silico* prediction tools have been used. Since most of the variations covered by SNP arrays are non-coding, we have used tools and databases providing annotation information on non-coding SNPs.

To prioritize variations, we have proceeded as follows: 1) RegulomeDB, a database that annotates SNPs with known and predicted regulatory elements in the intergenic regions of the H. sapiens genome, was used to analyze the regulatory effects of noncoding variants [26].

2) Top ranked SNPs (RegulomeDB score = 1) were then investigated using the GTEx portal to evaluate the association of potential functional variants with gene expression in breast mammary tissue [27] 3) SNPnexus [28] and VARAdb [15] databases were used to annotate variations and to retrieve regulatory features of the investigated SNPs while SNPinfo web server was used to predict the potential miRNA binding sites (https://snpinfo.niehs.nih.gov/cgi-bin/snpinfo/snpfunc.cgi).

### Statistical analysis

Comparison of allele frequencies of the candidate SNPs was performed using Chi-square test with one degree of freedom. Bonferroni’s adjustments were applied for the selected SNPs and a p-value< 0.05 was considered statistically significant.

### miRNAs pathways investigation

We determined the pathways that are regulated by miRNA using miR+Pathway (http://www.insect-genome.com/miR-pathway) that provide information regarding miRNA-target interactions.

## Results

In the current study, we have assessed linkage disequilibrium and haplotype diversity within high and moderate penetrance breast cancer susceptibility genes in the Tunisian population through the analysis of 387 SNPs identified in *BRCA1*, *BRCA2*, *STK11*, *PTEN*, *TP53*, *ATM*, *BRIP1*, *CHEK2* and *PALB2* genes.

We have also investigated the functional impact of the identified SNPs and selected putative functional variants that may contribute to disease susceptibility. Interpopulation variability between the Tunisian population and 8 other distinct populations of the 1000 genomes project was also assessed.

### Haplotype analysis

**Fig 1:**
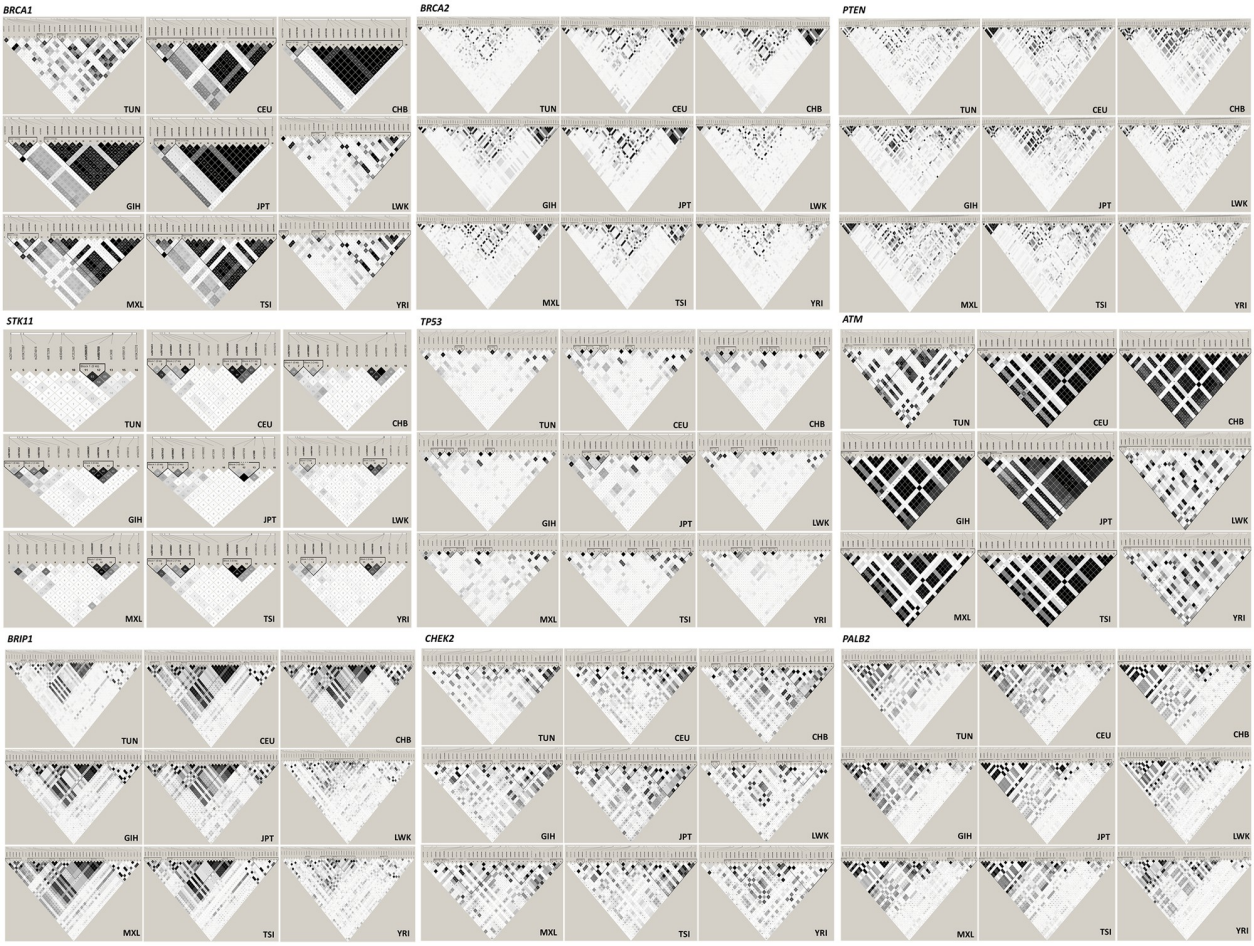
LD patterns and haplotype structure of high and moderate penetrance breast cancer susceptibility genes in the Tunisian population and 8 populations of the 1000 genomes project. LD blocks were constructed using Haploview program version 4.2. In each box the intensity of the shadow represents the increasing strength of LD. White blank boxes represent lack of LD while dark boxes signify strong LD patterns. Tagging SNPs are highlighted in bold.

Haplotype analysis was performed for each gene of interest. Linkage Disequilibrium (LD) profiles and haplotype structure within the Tunisian population and those of the 1000 genomes project are illustrated in Fig 1. In the Tunisian population, haplotype analysis revealed high levels of recombination across the investigated genes. In fact, most of the variations show low levels of LD and haplotype blocks are medium to small separated by recombination hotspots regions. The smallest blocks were observed in *BRCA2*, *PTEN*, *STK11* and *TP53* genes in favor of higher recombination rates in these genes compared to the others. When comparing haplotype structure of the characterized ethnicities we note that the Tunisian population tends to have LD patterns comparable to that observed in populations with African ancestries namely Yoruba in Ibadan, Nigeria and Luhya in Webuye, Kenya. However, the Tunisian population seems to have smaller haplotype blocks compared to all the other worldwide populations and this can be clearly observed in *BRCA1*, *BRCA2* and *ATM* genes. Particularly for *BRIP1* gene, LD patterns and haplotype blocks are more likely similar to those observed in American population with Mexican Ancestry. To assess the genetic diversity of the Tunisian population we have built a haplotype network for the major susceptibility genes *BRCA1* and *BRCA2*. Haplotypes used to construct the network are given in S1 Table.

**Fig 2:**
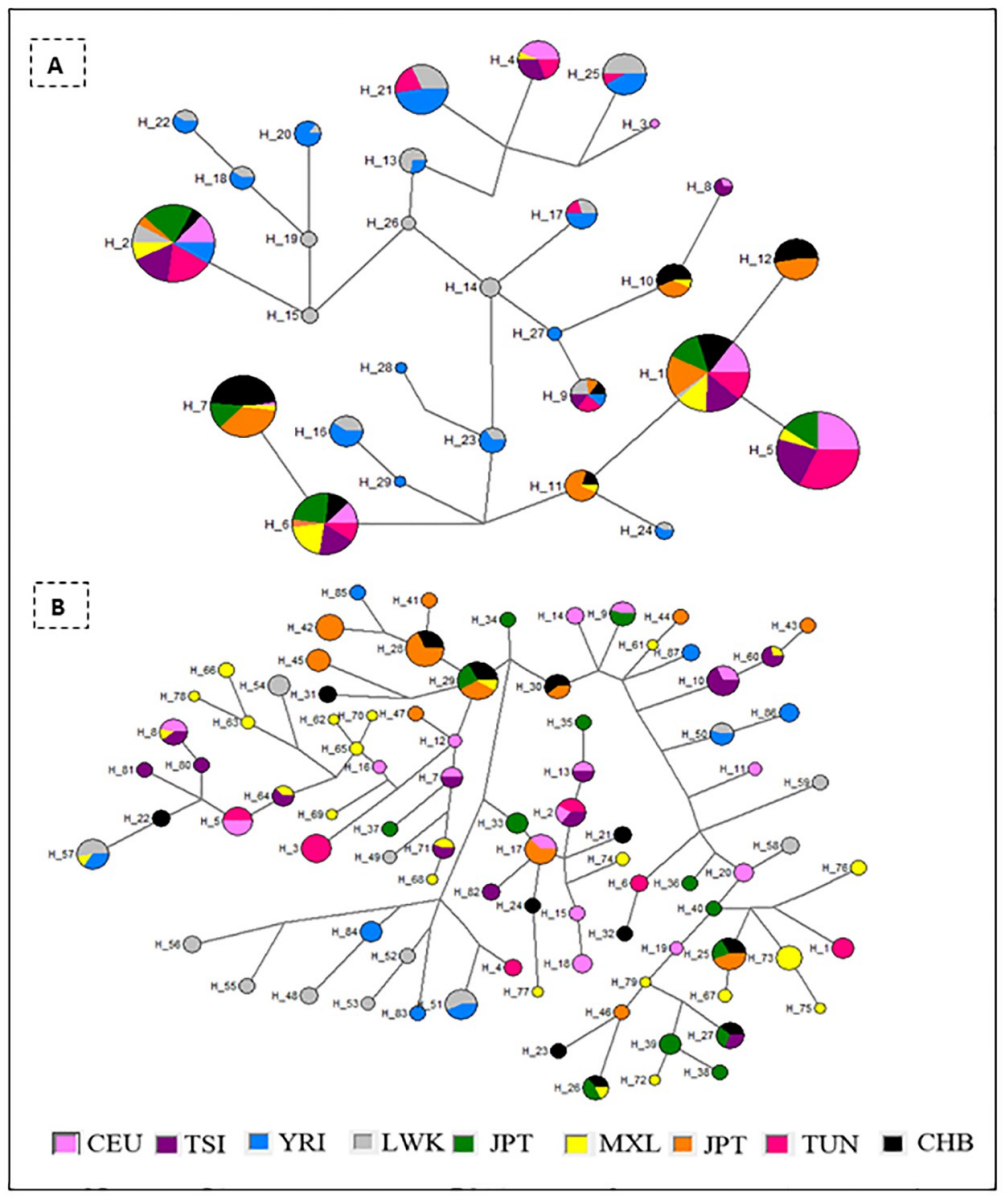
Median-joining haplotype network for BRCA1 (A) and BRCA2 (B) gene. The sizes of the circles are proportional to haplotype frequency and each color refer to a distinct population.

For the ***BRCA1*** gene a total of 29 haplotypes were analyzed. The H1 haplotype was the most common (n = 511) occurring in all analyzed populations except that from Yoruba in Ibadan (Fig 2). The second most common haplotype was H2 (n = 252) and was observed across all populations included in this study. As shown in Fig 2 all haplotypes identified in the Tunisian population are shared with more than one other population. Three haplotypes namely H17, H21, and H25 are specific to those with African ancestry TUN-LWK-YRI. Regarding ***BRCA2*** gene, 87 haplotypes were studied. The most frequent haplotype was H29 found in 4 populations CHB, GIH, JPT and MXL. Haplotypes H1, H3, H4, and H6 were found to be specific to the Tunisian population.

Furthermore, we have calculated allele frequencies and defined haplotype tagging SNPs (htSNPs) for each gene of interest (S2 and S3 Tables).

### Population structure and genetic diversity analysis

**Fig 3:**
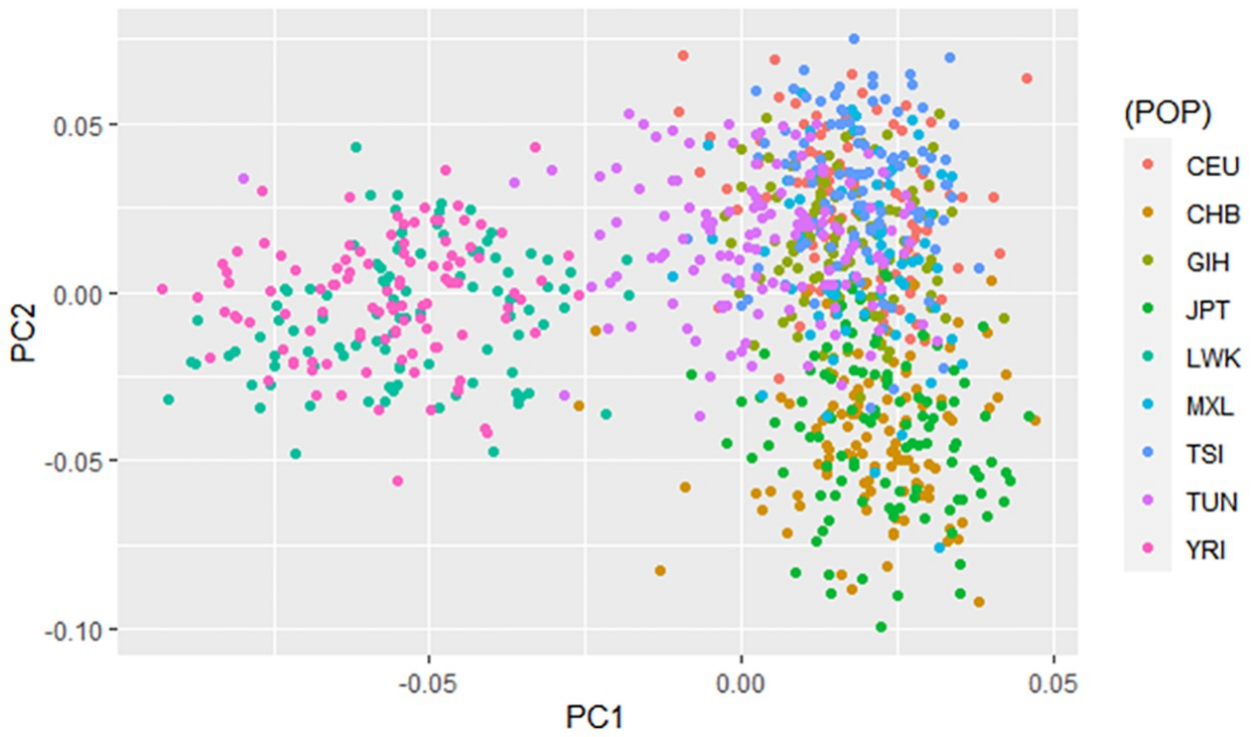
Principal component analysis of high and moderate penetrance susceptibility genes. The first two principal components (PCs) are plotted and coloured according to population ancestry.

To further investigate the genetic diversity of the Tunisian population and to assess its genetic ancestry, a PCA was performed. This latter showed that the Tunisian population is clustered with populations of European (CEU & TSI), South Asian (GIH), and Mexican ancestry (MXL) (Fig 3). The plot also clearly reveals that populations of African ancestry (LWK and YRI) are distinguished from non-African populations and form a distinct cluster.

Furthermore, we used Wright’s fixation index Fst to evaluate the genetic divergence among Tunisian and the other 8 worldwide populations (S1 Appendix). Fst values ranged from 0 to 0.496. The highest levels of differentiation were encountered in comparison with populations of African (YRI = 0.442 & LWK = 0.426) and East Asian (JPT = 0.469 & CHB = 0.364) ethnicity. These results support the findings of the PCA and indicate that the genetic background of the Tunisian population is closer to that of populations with non-African ancestry.

### Functional annotation

The vast majority of SNPs characterized from genotyping data are localized in untranslated regions. Given that non-coding elements can have diverse roles in the regulation of protein-coding genes, we performed functional annotation using RegulomeDB to identify potential functional variants. RegulomeDB uses a scoring system to classify variations. Lower scores indicate increasing evidence for a variant to be located in a functional region [26]. To prioritize candidate functional variants only top ranked ones, those belonging to category 1, were considered for further analysis. These variations are likely to affect binding and are linked to the expression level of a target gene. These SNPs were then explored in the GTEx database to evaluate their association with gene expression in breast mammary tissue. Additional regulatory features were also assessed using SNPNexus, VARAdb and SNPinfo web-based tools. A total of 28 potentially functional SNPs, with supporting evidence of regulatory functions were selected (Table 1).

**Table 1:** Putative functional SNPs identified in high and moderate penetrance breast cancer susceptibility genes.

Gene	Rs ID	RegulomeDB Score	GTEx-eQTL in Breast- Mammary tissue	Regulatory features	Putative altered miRNA binding sites
***BRCA1***	**rs1043284**	1d	*NBR2* (p-value = 0.000031) and *IFI35* (p-value = 9.3e-7)	Located in typical enhancers in breast epithelium tissue (ENCODE data)	hsa-miR-1321; hsa-miR-220c; hsa-miR-513a-5p; hsa-miR-513b; hsa-miR-556-5p; hsa-miR-659 (miRanda)
	**rs2271539**	1f	*NBR2* (p-value = 1.1e-20), CTD-3199J23.6 (p-value = 0.0000017), LINC00854 (p-value = 0.000025)	Located in typical enhancers in breast epithelium tissue (ENCODE data) and in regions with histone modifications with H3K4me3 mark (ENCODE &Roadmap data)	
	**rs1567832**	1d	*NBR2* (p-value = 1.2e-17), *CTD-3199J23*.*6 (p-value = 7*.*8e-7*), *RUNDC1 (p-value = 0*.*000015)*, LINC00854 (p-value = 0.000023)	Located in typical enhancers in breast epithelium tissue (ENCODE data)	
	**rs10840**	1f	*NBR2(p-value = 0*.*0000074)*, IFI35 (p-value = 0.0000012)		hsa-miR-33a, hsa-miR-33b, hsa-miR-450a, hsa-miR-450b-5p, hsa-miR-507, hsa-miR-544, hsa-miR-663b, hsa-miR-892a (miRanda); mmu-miR-466a-5p, mmu-miR-466b-5p, mmu-miR-466c-5p, mmu-miR-466e-5p (Sanger)
	**rs8176318**	1f	*NBR2 (p-value = 6*.*1e-64)*, *LINC00854 (p-value = 3*.*2e-8)*, *CTD-3199J23*.*6(p-value = 3*.*6e-8)*, *RND2* (p-value = 0.000016)	Located in open chromatin regions in fibroblast of mammary gland in primary cells (ENCODE data)	hsa-miR-1182, hsa-miR-149, hsa-miR-345, hsa-miR-544, hsa-miR-639 (miRanda); mmu-miR-540-5p (Sanger)
	**rs4793197**	1f	NBR2 (p-value = 1.1e-64), CTD-3199J23.6 (p-value = 1.1e-8), LINC00854 (p-value = 2.6e-8), RND2 (p-value = 0.000012)	Located in enhancers targeting *BRCA1*, *IFI35*, *VAT1*, *RUNDC1* genes in Breast Myoepithelial Primary Cells (Roadmap127 epigenome samples data)	
***BRCA2***	**rs206336**	1f	No significant eQTLs	Located in typical enhancers in breast epithelium tissue (ENCODE data) and in regions with histone modifications with H3K4me3 mark (ENCODE & Roadmap data) Located in open chromatin regions in breast tumors (TCGA data)	
	**rs206337**	1f	No significant eQTLs	Located in typical enhancers in breast epithelium tissue (ENCODE data) and in regions with histone modifications with H3K4me3 mark (ENCODE&Roadmap data) Located in open chromatin regions in breast tumors (TCGA data) and in breast epithelium tissue (ENCODE data)	
	**rs798273**	1b	No significant eQTLs	Associated with *CG030* gene expression in breast tumors (p-value = 0.000116) (PancanQTL data)	
***STK11***	**rs12488**	1d	*C19orf24* (p-value = 1.1e-18)	Located in super enhancers in breast epithelium tissue (ENCODE data) and in regions with histone modifications with H3K4me3 mark (ENCODE & Roadmap data) Associated with *C19orf24* expression (p-value = 3.66e-06) in breast tumors (PancanQTL data)	
***TP53***	**rs1050540**	1f	*EFNB3* (p-value = 0.000027)		hsa-miR-579 (miRanda)
	**rs1050541**	1a	No significant eQTLs		hsa-miR-1224-5p, hsa-miR-1255a, hsa-miR-1255b, hsa-miR-1265, hsa-miR-1303, hsa-miR-1321, hsa-miR-765, hsa-miR-768-5p (miRanda)
***ATM***	**rs228591**	1f	No Significant eQTLs	Associeted with *ATM* expression in breast tumors (p-value = 0.000362) (PancanQTL data)	
	**rs227073**	1f	No Significant eQTLs	Located in breast cancer-associated enhancer targeting ATM gene (DiseaseEnhancer data) Associated with *CUL5* expression (p-value = 0.000575) in breast tumors (PancanQTL data)	
***CHEK2***	**rs5752811**	1f	No Significant eQTLs	Located in super enhancers in Breast epithelium tissue (ENCODE data) Located in open chromatin regions in breast tumors (TCGA data) and in breast epithelium tissue (ENCODE data)	
***PALB2***	**rs11642434**	1f	*DCTN5 (p-value = 6*.*0e-17)*, *EARS2 (p-value = 5*.*9e-9)*, *CTD-2270L9*.*2* (p-value = 0.0000067)	Located in tumorigenesis-associated enhancer (EnDisease data) Associeted with *COG7* (p-value = 8.5e-09) and *DCTN5* (p-value = 4.54e-10) expression in breast tumors (PancanQTL data)	
	**rs6497674**	1f	*DCTN5* (p-value = 1.1e-16), *EARS2* (p-value = 2.4e-13)	Located in tumorigenesis-associated enhancer (EnDisease data) Located in open chromatin regions in breast tumors (TCGA data)	
	**rs1610**	1f	*DCTN5* (p-value = 1.8e-13), *EARS2* (p-value = 3.3e-16)	Located in tumorigenesis-associated enhancer (EnDisease data) Associated with *COG7* (p-value = 5.29e-09) and *DCTN5* (p-value = 1.3e-12) genes in breast tumors (PancanQTL data)	hsa-miR-136, hsa-miR-508-3p (miRanda); hsa-miR-181a-3p (TarBase)
	**rs152454**	1d	*DCTN5* (p-value = 1.6e-13), *EARS2* (p-value = 6.2e-17)	Located in tumorigenesis-associated enhancer (EnDisease data) Associated with *COG7* (p-value = 1.87e-09) and *DCTN5* (p-value = 1.13e-12) genes expression in breast tumors (PancanQTL data)	hsa-miR-125a-3p, hsa-miR-298, hsa-miR-556-5p, hsa-miR-610 (miRanda)
	**rs459894**	1f	DCTN5 (p-value = 3.1e-9), EARS2 (p-value = 0.0000085)	Located in tumorigenesis-associated enhancer (EnDisease data) Associated with *COG7* (p-value = 7.89e-08) and *DCTN5* (p-value = 4.7e-07) genes in breast tumors (PancanQTL data)	
	**rs426745**	1f	*DCTN5 (p-value = 1*.*7e-8)*	Located in tumorigenesis-associated enhancer (EnDisease data Associated with *COG7* (p-value = 4.03e-08) and *DCTN5* (p-value = 1.04e-06) expression in breast tumors (PancanQTL data)	
	**rs380618**	1b	*DCTN5* (p-value = 2.6e-9), *EARS2 (p-value = 0*.*0000011)*	Located in tumorigenesis-associated enhancer (EnDisease data) and in regions with histone modifications in breast epithelium tissue with H3K4me3 mark (ENCODE& Roadmap) Associated with *COG7* (p-value = 6.4e-06) and DCTN5 (p-value = 9.18e-06) genes in breast tumors (PancanQTL data)	
	**rs120963**	1f	*DCTN5 (p-value = 6*.*0e-17)*, *CTD-2270L9*.*2 (p-value = 0*.*0000067)*, *EARS2 (p-value = 5*.*9e-9)*	Located in tumorigenesis-associated enhancer (EnDisease data) and in regions with histone modifications in breast epithelium tissue with H3K4me3 mark (ENCODE& Roadmap data) Predicted to strongly affect the binding of the TFs TP53, STAT3 and CEBPG (ENCODE-Motif data); Associated with COG7 (9.41e-10) and DCTN5 (8.42e-13) genes expression in breast tumors (PancanQTL data) Located in enhancers targeting *PALB2*, *NDUFAB1*, *UBFD1*, *EARS2*, *DCTN5 and COG7 genes* in Breast Myoepithelial Primary Cells (Roadmap 127 epigenome samples data)	
	**rs420259**	1f	*DCTN5 (p-value = 3*.*8e-19)*	Located in tumorigenesis-associated enhancer (EnDisease data) Associated with *COG7* (p-value = 7.17e-05) and DCTN5 (p-value = 7.71e-14) expression in breast tumors (PancanQTL data)	
	**rs8043812**	1f	*DCTN5* (p-value = 3.2e-13)	Located in tumorigenesis-associated enhancer (EnDisease data) Associated with DCTN5 (p-value = 4.84e-18) expression in breast tumors (PancanQTL data)	
	**rs26760**	1f	*DCTN5 (p-value = 1*.*7e-36)*, *EARS2* (p-value = 0.000044)	Located in tumorigenesis-associated enhancer (EnDisease data) Associated with COG7 (p-value = 2.46e-05) and DCTN5 (p-value = 7.33e-27) expression in breast tumors (PancanQTL data)	hsa-miR-1236, hsa-miR-1260, hsa-miR-197, hsa-miR-220b (miRanda)
	**rs35635**	1f	*DCTN5* (p-value = 3.4e-37), *EARS2 (p-value = 0*.*000016)*	Located in tumorigenesis-associated enhancer (EnDisease data) Associated with COG7 (p-value = 4.44e-06) and DCTN5 (p-value = 1.68e-29) expression in breast tumors (PancanQTL data)	
	**rs26766**	1f	*DCTN5 (p-value = 5*.*8e-14)*	Located in tumorigenesis-associated enhancer (EnDisease data) Associated with COG7 (p-value = 000101) and DCTN5 (p-value = 5.85e-11) expression in breast tumors (PancanQTL data)	

In the *BRCA1* gene, 6 putative functional variants were detected (Table 1). All these variations are associated with the expression levels of *NBR2*, a non–protein coding gene that resides adjacent to *BRCA1*. Furthermore, the 3 first SNPs (rs1043284, rs2271539, rs1567832) were located in typical enhancers and among these variants rs2271539 lies in H3K4me3 histone modification region. We have also found that rs8176318 sets in an open chromatin region while rs4793197 was located in enhancers targeting *BRCA1*, *IFI35*, *VAT1* and *RUNDC1* genes. Additional *in silico* analysis showed that rs1043284, rs10840, rs8176318 are predicted to alter the binding sites of several miRNAs including hsa-miR-149, hsa-miR-544 and hsa-miR-639. For *BRCA2*, 3 candidate variants were selected namely rs206336, rs206337 and rs798273. rs206336 and rs206337 were located in typical enhancers, in accessible chromatin regions and lie also in the H3K4me3 histone modification region. For *STK11*, rs12488 was associated with the expression levels of *C19orf24* in both breast mammary tissue and breast tumors. It also sets in super enhancers and in chromatin regions with H3K4me3 mark.

Considering *TP53* gene, rs1050540 was predicted to alter the binding sites of hsa-miR-579 while rs1050541 seems to affect the binding sites of 8 distinct miRNAs such as hsa-miR-1224-5p and hsa-miR-1265. For the *ATM* gene, rs228591 was found to be associated with *ATM* expression in breast tumors (p-value = 0.000362) and rs227073 was located in breast cancer-associated enhancer targeting *ATM* gene. In *PALB2*, 13 candidate functional variants were selected (Table 1). These latter were associated with gene expression of *DCTN5* and were located in tumorigenesis-associated enhancers.

*PALB2*_rs6497674 was located in open chromatin regions, whereas rs380618 was located in chromatin regions with H3K4me3 mark. Interestingly, rs120963 was located in a tumorigenesis-associated enhancer, associated with histone mark H3K4me3 and was predicted to strongly affect the binding of 3 TFs TP53, STAT3 and CEBPG. It was also located in enhancers targeting *PALB2*, *NDUFAB1*, *UBFD1*, *EARS2*, *DCTN5* and *COG7* genes.

### Allele frequency comparison between different ethnic groups

The pairwise comparison of allele frequencies of the candidate SNPs between the Tunisian population and those of the 1000 genomes project revealed significant differences in allele frequencies either with Africans, Europeans, Americans, or Asians (S4 Table). Taking the examples of rs1043284 and rs10840 in the *BRCA1* gene, significant differences were observed with all populations except those from European ancestry. Nevertheless, for *PALB2* gene allele frequencies of rs11642434, rs120963, rs420259, rs26760, rs35635 and rs26766 were significantly different from those observed in European ethnic populations. Moreover, compared to African populations, significant differences in allele frequencies were found for rs2271539, rs1567832, rs8176318, rs4793197 in *BRCA1*, rs1050541 in *TP53*, rs5752811 *in CHEK2* and rs459894, rs426745, rs120963 in *PALB2* gene. Furthermore, when considering all 1000 genomes project ‘s populations merged together we note that *BRCA2*_rs206336, *BRCA2_*rs206337, *STK11*_rs12488, *ATM*_rs228591 and *ATM* _rs227073 had allele frequencies specific to the Tunisian population.

### miRNAs pathways investigation

In our study, several SNPs were found to alter the binding sites of miRNAs. Some of these miRNAs are involved in important pathways, such as the PI3-Akt signaling pathway, Ras signaling pathway, MAPK signaling pathway, P53 signaling pathway…ect (S5 Table). Moreover, several miRNAs involved in cancer pathways were identified. Interestingly, hsa-miR-136, hsa-miR-639 and hsa-miR-298 were found to regulate several cancer pathways such as breast, colorectal, pancreatic, gastric, and prostate cancer.

## Discussion

Considerable progress has been made towards the understanding of the genetic basis of breast cancer [29]. So far, Genome-wide association studies have successfully identified more than 170 loci associated with breast cancer risk [14]. These risk-associated SNPs are usually not the causal ones, but they are in high LD with functional SNPs causative of the investigated phenotype [17, 29]. To identify functional SNPs, it is essential to consider the haplotype structure of the studied population. This has been facilitated by genome sequencing projects including the International HapMap Project and the 1000 Genomes Project that have produced high quality genotyping data in large sample sizes of diverse human populations. This helps in identifying functional SNPs by using population-specific haplotype structures [17]. Unfortunately, North African populations are not represented in these projects hence the need to characterize the genetic risk loci and haplotype structure of susceptibility genes to better understand the GWAS findings. The clinical insights derived from GWAS findings have been limited given that most of the risk -associated SNPs have been mapped to non-coding regions and the interpretation of their functional impact remains challenging [13]. In the current report, we have characterized the LD patterns and haplotype blocks of high and moderate penetrance susceptibility breast cancer genes. We have also assessed the functional impact of the studied SNPs. Characterization of haplotype structures showed differences in LD patterns between genes with high levels of recombination and haplotype blocks of moderate to small size. These results are predictable given the fact that the African population is older than the other populations, in which there have been more recombination events resulting in smaller blocs [30]. These findings are also in accordance with previous reports where populations with African ancestry were found to have smaller blocks than non-African populations [17, 31]. In addition, variation in LD patterns between genes are expected. In fact, it was shown that the strength and the distance over which LD extends vary along chromosomes and from one region of the genome to another [32, 33]. Several factors seem to shape patterns of diversity and LD in the human genome. These included population size, genetic drift, selection, demographic factors and variable rates of mutation and recombination. We have also determined tagging SNPs constituted with the most informative markers that can be used in upcoming large-scale breast cancer association studies in Tunisia. In the current report tagging SNPs were used to construct a haplotype Network for *BRCA* genes that included genotypic data from Tunisia and from 8 other human populations. The most frequent haplotypes identified for each gene are probably the ancestral ones from which all others have derived. Moreover, Tunisian haplotypes showed a heterogeneous distribution, some are shared with different populations and others are unique to the Tunisian population. These results reflect the mosaic genetic structure of the Tunisian population which may explain the genetic heterogeneity of breast cancer in Tunisia. This observation was further confirmed with PCA and Fst estimation. In fact, our findings have revealed that the Tunisian population tends to have a mixed origin divergent from that of African populations. In fact, it was found to be clustered with populations of European, South Asian, and Mexican ancestry and showed a high level of differentiation with those of African ethnicity. This could be explained by migratory waives that occurred in Mediterranean countries. Indeed, throughout its history, Tunisia has witnessed invasions and several migratory flows in prehistoric and historic periods which contributed to its genetic diversity [34, 35]. Our findings are in accordance with previous studies investigating the genetic structure of the Tunisian population. In fact, it was demonstrated that the Tunisian population has an admixed genetic structure with prevalence of the Eurasian lineages, followed by the Sub-Saharan and North African ancestries [36, 37]. Furthermore, we have explored the functional impact of the studied SNPs and have selected 28 putative functional variants. These variations were located in transcriptional regulatory regions such as enhancers, TFs, accessible chromatin regions and miRNAs binding sites. An interesting variant was detected in *BRCA1* gene rs8176318 that was associated with *NBR2* gene expression in mammary tissue and was predicted to alter the bindings of 6 distinct miRNAs. *NBR2* is a non–protein coding gene localized adjacent to the *BRCA1* gene. The two genes are separated by only 218 base pairs and their transcription is divergent. Some studies have revealed that *NBR2* and *BRCA1* genes may in fact, be reciprocally regulated. In a number of cell lines, higher levels of *NBR2* expression are correlated with low *BRCA1* levels. This could reflect competition between the promoters for RNA polymerase II and also suggests that activation of the *NBR2* promoter could lead to decreased *BRCA1* expression [38]. In light of these observations, we may hypothesize that SNPs found to be associated with increased *NBR2* expression could be associated with downregulation of *BRCA1* and hence with breast cancer risk. Nevertheless, and in a more recent study, Xiao et al. have shown that *NBR2* encodes a long non-coding RNA and suppresses tumor development through regulation of adenosine monophosphate–activated protein kinase (AMPK) activation which means that downregulation of *NBR2* gene may also predispose to cancer [39]. In a previous genome wide SNP genotyping study conducted by our group, a putative functional SNP “rs9911630” was also found to be associated with the expression levels of *NBR2*. This variant, and in addition to its functional impact contributed very significantly to the genetic variability between African and non-African populations [12]. *BRCA1*_rs8176318 that we are describing here has been reported as being associated with familial breast and ovarian cancer in Thai population and with triple negative disease among African American women [40]. It was also found to be associated with decreased *BRCA1* expression in breast cancer cell lines, and with greater likelihood of having stage IV breast cancer [41]. It is well established that allele frequency varies between populations worldwide leading to variation in disease susceptibility. *BRCA1*_rs8176318 was identified with a relatively high frequency in Tunisian healthy individuals (27.6%). Comparison of allele frequency revealed statistically significant differences with African populations, YRI (12.5%), and LWK (11.1%), with respective p-values of 0.0004388 and 0.0001243. These results showed that the Tunisian population may have an increased breast cancer risk compared to populations from African ancestries. Likewise, among putative miRNAs disrupted by this variant, hsa-miR-149 was reported to be downregulated in breast tumors and it is usually recognized as a tumor suppressor with reduction in several cancers [42]. Furthermore, we have found that hsa-miR-639 regulates several cancer pathways including breast, endometrial, pancreatic, and colorectal cancer. It was also demonstrated that the up-regulation of this miRNA contributes to breast cancer invasion and metastasis [43]. Another interesting variant was identified in *PALB2* gene rs120963. This SNP was predicted to strongly affect the binding of three TFs TP53, STAT3 and CEBPG. The tumor suppressor p53 is a DNA-binding transcription factor that plays a critical role in preventing cancer progression. Altering the binding of this TF may promote cancer growth [44]. This variant was also located in enhancers targeting *PALB2*, *NDUFAB1*, *UBFD1*, *EARS2*, *DCTN5* and *COG7* genes in breast myoepithelial primary cells. Enhancers can activate gene transcription by recruiting transcription factors, chromatin remodeling factors and RNA Polymerase II [45]. Single nucleotide variants in these regulatory elements might affect transcription factor bindings, and in turn, make an essential contribution to disease progression by altering the expression of the target genes [46]. This variant sites also in chromatin regions with the H3K4me3 mark. This latter is one of the epigenetic modifications to histone proteins [47]. that is widely enriched at promoters and transcriptional start sites (TSS) of highly expressed genes [48]. It was shown that H3K4me3 interacts with chromatin remodelers and promotes recruitment of basic transcription factors to facilitate transcription activation [47, 49]. Along these lines, variations in tumor suppressor genes occurring in H3K4me3 domains may alter the accessibility of genes for transcription and promote cancer development and progression. Previous studies investigating *PALB2*_ rs120963 have reported increased breast cancer risk associated with this variant [50, 51]. Comparison of allele frequency that we have conducted showed statistically significant differences between the Tunisian population and those of European and African ancestries. Several other candidate functional SNPs located in open chromatin regions were identified such as *BRCA2_rs206336* and *CHEK2_rs5752811*. Accessible chromatin represents the transcriptionally active state of each gene. It displays lower nucleosomal density or even nucleosome-free regions and captures more than 90% of TFs target regions [52, 53]. Variations identified within these regions may therefore affect gene expression by altering TFs bindings sites. These putative functional SNPs that we have characterized should be prioritized in upcoming association studies since they may act as genetic markers of cancer risk. In fact, they could serve to develop a polygenic risk score (PRS) specific to the Tunisian population which will help to improve breast cancer screening in Tunisian individuals. This PRS constitute an estimate of an individual’s genetic susceptibility to a trait or disease, estimated according to their genotype profile and relevant GWAS data [54]. In fact, combinations of known risk-associated variants might be useful tools in identifying individuals with relatively higher risk among general populations for targeted cancer prevention [55, 56]. Indeed, several previous studies have described a higher PRS among women diagnosed with breast cancer when compared to healthy individuals [57]. The correlation between the PRS and the genetic liability to disease has led to its routine application in biomedical research [54]. Implementing polygenic testing in the Tunisian population is of keen interest. It may help to resolve part of the missing heritability and to identify at risk women with uninformative genetic test results. This will revolutionize health services by providing personalized risk assessments within population breast screening programs [57].

## Conclusions

In the current report characterization of the LD profiles and haplotype structures showed some substantial differences between the studied genes and also among populations. This underscores the need to characterize each locus of interest prior to association studies. Haplotype distribution was also heterogeneous, reflecting the mosaic structure of the Tunisian population. We have also provided an overview of the potential functional variants that could be associated with breast cancer risk in the Tunisian population and that may serve to develop a PRS leading to better disease prevention.

## Supporting information

S1 Appendix The fixation-index (Fst) analysis results. (XLSX)

S1 Table Aligned haplotypes with frequency >1% used for haplotype network construction. (XLSX)

S2 Table Allele frequencies of the investigated SNPs in the Tunisian population. (XLSX)

S3 Table Haplotype tagging SNPs of the high and moderate penetrance breast cancer susceptibility genes in the Tunisian population. (XLSX)

S4 Table Comparison of Allele frequencies of the 28 putative functional variants between the Tunisian population and 8 worldwide populations of the 1000 genomes project. (XLSX)

S5 Table Pathways regulated by disrupted miRNA. (XLSX)
